# Apparent strength versus universality in glasses of soft compressible colloids

**DOI:** 10.1038/s41598-018-35187-9

**Published:** 2018-11-14

**Authors:** Ruben Higler, Joris Sprakel

**Affiliations:** Physical Chemistry and Soft Matter, Wageningen University & Research, Stippeneng 4, 6708 WE Wageningen, The Netherlands

## Abstract

Microgel colloids, solvent swollen hydrogel particles of microscopic size, are in osmotic equilibrium with their surroundings. This has a profound effect on the behaviour of dense solutions of these polymeric colloids, most notably their ability to swell and deswell depending on the osmotic pressure of the system as a whole. Here we develop a minimal simulation model to treat this intrinsic volume regulation in order to explore the effects this has on the properties of dense solutions close to a liquid-solid transition. We demonstrate how the softness dependent volume regulation of particles gives rise to an apparent change in the fragility of the colloidal glass transition, which can be scaled out through the use of an adjusted volume fraction that accounts for changes in particle size. Moreover, we show how the same model can be used to explain the selective deswelling of soft microgels in a crystalline matrix of harder particles leading to robust crystals free of defects. Our results not only highlight the non-trivial effects of osmotic regulation in governing the apparent physics of microgel suspensions, but also provides a platform to efficiently account for particle deswelling in simulations.

## Introduction

Colloidal suspensions exhibit a marked slowing down of structural relaxation processes when the particle volume fraction is increased^1–6^. In analogy to the dynamical slowing down in molecular liquids upon quenching the temperature, given that crystallization is suppressed, this is often denoted as a colloidal glass transition. The colloidal glass transition exhibits some striking similarities to its molecular equivalent, such as the emergence of inhomogeneous, collective dynamics^4,5,7^ and anomalies in the vibrational density-of-states^8–10^. For hard sphere colloids, interacting solely by volume exclusion, the liquid viscosity grows steeply around a particle volume fraction *ϕ* = 0.58, which is identified as the glass transition point *ϕ*_*g*_, while undergoing a solid-liquid transition at slightly higher packing fractions due to jamming^11–15^. The steep growth in viscosity around *ϕ*_*g*_, predicted to diverge according to mode-coupling theory^16^, signals the drastic slowing down of structural relaxations over a very small window of volume fraction. Such a steep change in the dynamics with small changes in the experimental control parameter is denoted as a fragile liquid, the colloidal equivalent of the Angell classification of glass fragility^17–19^. A fragile liquid is characterized by a superexponential growth of the viscosity with *ϕ* upon approaching *ϕ*_*g*_. Seminal experiments on colloidal microgels have shown that this fragile transition for hard spheres can give way to one in which the growth of viscosity with packing density becomes much more gradual by increasing the softness of the colloids^20^. For these microgel suspensions, it was found that sufficiently soft colloids exhibit a purely exponential growth of relaxation time with *ϕ*, which was denoted as a strong glass former in analogy to the limiting Arrhenius-scenario in the molecular world^17–19^. This observation triggered the fascinating possibility that the diversity in glass transitions at the molecular scale may be emulated in the colloidal domain using the particle softness as a tuning knob. However, the interpretation of these observations are still a topic of debate^21,22^.

While the possibility of an analogy between glass fragility in the molecular and colloidal worlds is appealing, it also raises some unanswered questions. Most importantly, since the origins of fragility and strength remain incompletely understood in both molecular and colloidal glasses, mapping one problem onto the other does not necessarily advance our insight. Moreover, since the mechanisms are not fully clear for both cases, it is also not directly obvious that fragility at both scales is governed by the same principles. It may be possible that apparent similarities are in fact the result of very different microscopic physics. Yet, recent publications hint that a universal explanation may be on the horizon; for metallic glasses, it was proposed that the softness of the interatomic potential controls the fragility of the vitrification process^23^, while a recent publication of our group has shown a quantitative theoretical approach to explain apparent fragility changes in colloidal glasses based on their compressibility, i.e. softness with respect to deswelling^21^. Focussing on the colloidal domain, which is the aim of this manuscript, the previously developed phenomenological mean-field model explores the effect of osmotic deswelling of particles on the perceived changes in dynamics^21^. By accounting for the deswelling of particles in response to the osmotic pressure of the bath the model predicts a strong non-linearity in the relationship between the true volume fraction *ϕ* and the apparent packing fraction *ζ*. The latter is the packing fraction the suspension would have if no deswelling takes place and is often the parameter used in experimental studies^20,24–26^. This softness-dependent non-linearity causes changes in the shape of the curve describing the slowing down of the dynamics as a function of the experimental control parameter *ζ*, with respect to that of the actual parameter that governs the dynamics: *ϕ*. As a consequence, the model suggested that fragility transition in soft colloidal systems are merely apparent ones due to the use of a control parameter that does not govern the microscopic dynamics in the suspension. The realisation that particle deswelling can cause apparent changes in the steepness of the glass transition was also reached recently after experimental studies on charged microgels^25^.

This result invokes the idea that no changes in physics underlie the apparent changes in fragility in colloidal system, but that it is merely an apparent effect due to a lack of knowledge of the real volume fraction of the system at a given particle number density. If this is true, one may argue that softness does not break the universality of the slowing down of relaxations with the appropriate state variable *ϕ* and a link with molecular fragility is lost. To resolve this issue, new methods are required in which no essential features of the system, such as a universal slowing down with *ϕ*, have to be assumed a-priori. Unfortunately, our previous analytic theory relied exactly on this assumption^21^ and the validity of the hypothesis above cannot be tested based on the results of this theory alone. Ideally, we would like to be able to explore the validity of observed fragility changes in soft colloidal systems without the necessity of making such simplifications.

In addition to the uncertainty of the microscopic mechanisms that control fragility transitions, it also remains unclear what the connection between the fragility of a liquid and its propensity to exhibit dynamical inhomogeneities is. Dynamical heterogeneities are a characteristic hallmark of the glass transition^4,7^, where the glass exhibits small pockets of few to hundreds of particles, in which the local mobility differs significantly from that of the ensemble mean. For molecular glass formers it is common to presume that the fragility of the glass transition is intimately linked to the emergence of cooperative motion, leading to inhomogeneous dynamics^27,28^. However, the validity of this argument remains under debate^29^, as does the causality of these two features. For colloidal systems, no in-depth exploration exists that unambigously connects the fragility of the glass transition and the emergence of clusters of cooperatively moving particles. However, experiments reveal a hint that such a connection may exist. A comparison between the behavior of glasses formed from soft microgels versus incompressible hard spheres, which exhibit strong and fragile glass transitions, respectively, as evidenced in other publications, seem to indicate that the glass of soft particles shows significantly more homogeneous dynamics as compared to their incompressible counterpart^30^. If the link between fragility and dynamical heterogeneity persists up to the colloidal scale, it could enable an in-depth study of the causality of this connection.

In this paper we employ a dynamical Monte Carlo simulation model to explore fragility transitions in two-dimensional glasses formed from soft and compressible disks. We show how the previously postulated fragility changes with particle compressibility can be recovered, without requiring the assumption of a universal slowing down or equation of state. Our results show how the liquids of soft colloids exhibit apparent fragility transitions in the parameter *ζ,* but when the data is replotted against the true volume fraction *ϕ* a universal mastercurve is obtained independent of softness. This implies that fragility changes with particle softness in colloidal systems are only apparent ones, which do not involve any changes in the microscopic physics of the system. This is further supported by the fact that also the extent of dynamical heterogeneities can be collapsed using the true volume fraction as the state variable. Finally, we illustrate how the simulation method we adopt here to account for particle deswelling can also be used to explore other features found in soft suspensions, such as the selective deswelling of impurities in colloidal crystals due to a softness mismatch.

## Particle Compressibility in Dynamic MC

To explore the effects of particle compressibility without requiring a-priori assumptions regarding the equation-of-state and the kinetic mastercurve, as required in our previous theoretical efforts, we employ a dynamical Monte Carlo model in which these effects emerge from a minimal model. We study vitrification in a two-dimensional system of bidisperse disks composed of *N* = 2000 discs of initial radii *a*_0,*L*_ = 0.7*σ* and *a*_0,*S*_ = 0.5*σ*, at a 1:1 number ratio, in a square box with periodic boundaries. To parameterize the model, we start from the conventional approach to simulate soft spheres, which is a simple power-law contact potential:

1 $${U}_{ij}(r)=\varepsilon \,{(\frac{{a}_{i}+{a}_{j}}{r})}^{6}$$

in which *ε* is the overlap energy at particle contact. While such a soft-sphere potential has been used to explore a wide variety of effects in soft sphere systems, such as vitrification and crystallisation, it does not capture the particle compressibility. If particles are highly compressible, the sizes *a*_*i*,*j*_ are no longer constants and will depend on the particle concentration and the local packing structure. Such osmotic deswelling has been extensively evidenced in experimental systems, for example microgel colloids or star polymers^31–34^.

To introduce compressibility of the particles into the Monte Carlo model, we consider the free energy of isotropic particle compression for soft compressible spheres swollen with a solvent, e.g. microgels or star polymers. Volume changes are described by the swelling ratio *α*_*i*_ = *a*_*i*_/*a*_0,*i*_, where *a*_0,*i*_ is the equilibrium swollen radius of particle *i* in the dilute limit, where osmotic forces are negligible. For the sake of simplicity, we presume that the swelling energy *W* is parabolic around its minimum at *a*_0,*i*_ for small changes in particle size. However, in reality particles exhibit a finite compressibility; upon strong compression most solvent is expelled from the polymeric objects and the particle compressibility effectively vanishes. To capture these effects we use a finite-compressibility parabolic form:

2 $${W}_{i}=\frac{\kappa {(1-{\alpha }_{i})}^{2}}{\frac{{\alpha }_{i}}{{\alpha }_{c}}-1}$$

in which *α*_*c*_ set the limiting degree of deswelling where the particle has expelled most solvent and becomes incompressible. The energy scale *κ* controls the effective compressibility of the particles, and is thus the softness parameter is this study.

For each monte carlo trial move, we either translate a randomly selected particle by some small distance *δr*, drawn from a Gaussian distribution with a zero mean and a width proportional to 1/*a*_*i*_^35^, or we perform a move in which the size of a randomly selected particle is updated *a*_*i*_(*t*) = *a*_*i*_(*t* − *δt*) + *δa*, in which $\delta a\ll {a}_{0}$. The effective time t* in the simulations is normalized to the acceptance rate of translation moves in accordance with previous studies^35,36^. Translation and (de)compression moves are rejected or accepted by evaluating the Boltzmann probability of the total energy defined as:

3 $$E=\sum _{i}^{N}\,\sum _{j}^{N}\,{U}_{ij}+\sum _{i}^{N}\,{W}_{i}$$

In this way, structural relaxation by translation *and* osmotic (de)swelling, both occurring in systems of compressible particles, can be accounted for on a coarse-grained level.

It is our aim here to explore how compressibility effects the nature of the slowing down of relaxations in fluids of soft colloids. To this end, we choose a constant value of the contact energy *ε*/*k*_*B*_*T* = 5 and collapsed particle size *α*_*c*_ = 0.25 and vary the compressibility constant *κ*. In some sense, this is equivalent to varying the Poisson’s ratio *ν* of the particles; for *κ* = ∞, the particles are incompressible (*ν* = 0.5), e.g. describing emulsion droplets, while finite values of *κ* give a phenomenological description of compressible particles, *ν* < 0.5 such as microgel colloids or star polymers.

In our previous analytical theory we proposed that the origin of fragility transitions lie in the fact that osmotic deswelling of soft particles causes a non-linear relationship between the real volume fraction and the extrapolated volume fraction^21^. In this paper we study the glass transition of soft colloids in two dimensions and define the real area fraction as:

4 $$\varphi =\frac{\pi }{A}\,\sum _{i}^{N}\,{a}_{i}^{2}$$

where *A* = *L*^2^ is the area of the square simulation box. Since in experiments, also in three-dimensions, it is often not possible to obtain the size of each particle at the relevant volume fractions, many researchers make use of an extrapolated packing fraction that is defined as:

5 $$\zeta =\frac{\pi }{A}\,\sum _{i}^{N}\,{a}_{i,0}^{2}$$

which presumes that the particles retain their original size irrespective of the particle concentration. This is valid for incompressible disks where $\varphi \equiv \zeta$, but fails for soft particles as these can exhibit pronounced deswelling such that *ϕ* < *ζ*. In fact, deswelling can be so strong if the particles are sufficiently soft and the osmotic pressure sufficiently high, that the glass transition is moved from *ϕ*_*g*_ = 0.58 for hard disks to effective packing fractions $\zeta \gg 1$.

## Results and Discussion

Accounting for osmotic deswelling has a pronounced effect on the properties of the simulated colloidal glasses. The fact that osmotic deswelling is relevant in experimental systems, in particular for microgels and star polymers, has been confirmed extensively in previous studies, both experimentally^25,26,31,33,37–44^ and theoretically^45–47^. Here we account for compressibility to first order by introducing an energy associated with deswelling which takes the form of a quadratic finite-compressibility equation for each particle. We note that we have previously shown using analytical theory^21^ that the exact shape of the osmotic deswelling curve with respect to the particle density does not change the underlying physics, but only the absolute values of the packing fraction where deviations from the incompressible case occur. As it is our aim here to explore the concept of how particle compressibility effects fragility and dynamical heterogeneity in colloidal glasses, we use this simple form. However, our simulation approach can be readily adapted to different forms for the osmotic energy *W*, e.g. to describe a specific system in more microscopic detail.

**Figure 1:**
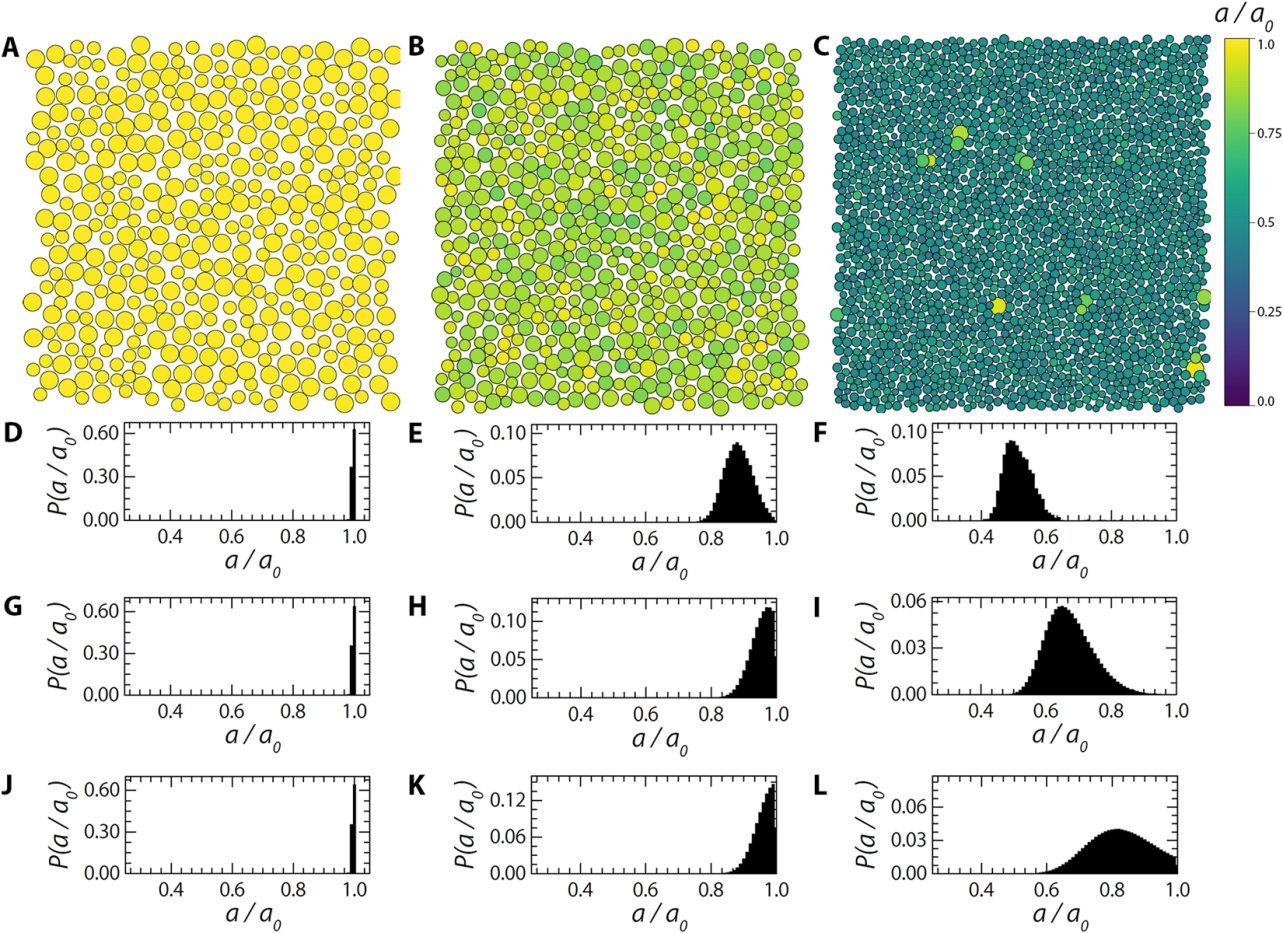
(**A**–**C**) Visual representation of two-dimensional glasses of compressible particles where the particles are colour-coded according to their relative deswelling ratio *a*/*a*_0_ as indicated by the colour bar, for *κ* = 50000 and *ζ*/*ζ*_*g*_ = 0.59 (**A**), *κ* = 500 and *ζ*/*ζ*_*g*_ = 0.58 (**B**) and *κ* = 50 and *ζ*/*ζ*_*g*_ = 0.59 (**C**). (**D**–**L**) Distribution of relative particle sizes, illustrating the extent of osmotic deswelling as a function of compressibility scale *κ* for *κ* = 50000 and *ζ*/*ζ*_*g*_ = 0.59 (**D**), 0.23 (**G**), 0.10 (**J**), *κ* = 500 and *ζ*/*ζ*_*g*_ = 0.58 (**E**), 0.23 (**H**), 0.06 (**K**) and *κ* = 50 and *ζ*/*ζ*_*g*_ = 0.59 (**F**), 0.23 (**I**), 0.10 (**L**).

We first explore how particle compressibility influences the size distribution of particles in the glass as a function of packing fraction. For rigid and largely incompressible particles, *κ* = 50000, we find, as expected, that the bidisperse population remains bidisperse even at high concentrations (Fig. 1A) and that the distribution of relatively deswelling degrees *P*(*a*/*a*_0_) is sharply peaked at 1 (Fig. 1D,G,J), indicative of little to no osmotic volume regulation up to the highest density explored here.

**Figure 2:**
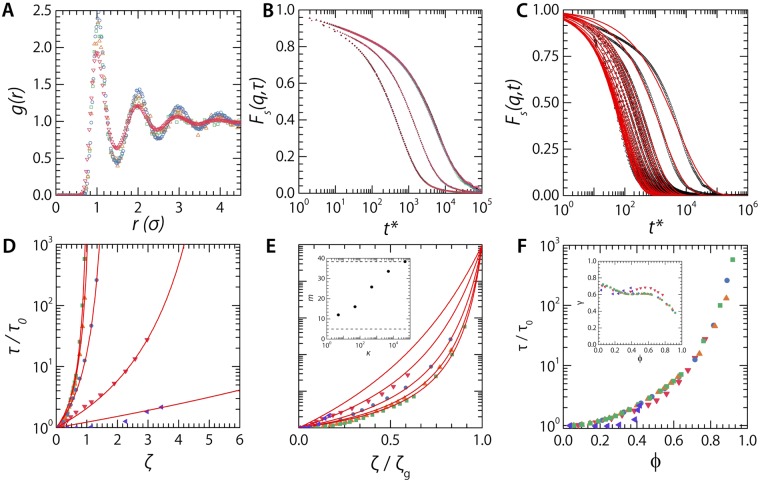
(**A**) Radial distribution function, *g*(*r*), calculated for simulation snapshots at *ζ* = 1.15 for *κ* = 5, 50, 500, 5000, & 50000 (left triangles, down triangles, circles, up triangles, squares). (**B**) *F*_*s*_(*q*, *t*) from triplicate repeats and the original simulation for *ζ* = (from left to right) 0.646, 0.769 & 0.86. (**C**) Self-intermediate scattering functions *F*_*s*_(*q*, *t*) from simulations (symbols) and fitted as described in the text (lines) for *q* = 5.2 *μm*^−1^, *κ* = 50000 and *ζ* = (from left to right) 0.0581, 0.103, 0.149, 0.192, 0.232, 0.258, 0.287, 0.322, 0.363, 0.413, 0.474, 0.534, 0.568, 0.605, 0.646, 0.769, 0.860, 0.930 & 1.010. (**D**) Terminal structural relaxation time *τ* as a function of the effective packing fraction *ζ* and (**E**) the same data in an Angell representation, where *ζ*_*g*_ is defined as the condition where log(*τ*/*τ*_0_) = 5. **Inset**: Kinetic fragility index *m* as a function of osmotic deswelling energy *κ*, dotted line at *m* = 5 gives the limiting fragility for a purely strong glass, dotted line at *m* = 38 that for the most fragile glass in these simulations. (**F**) The same data plotted as a function of *ϕ*, as calculated according to equation 4. Inset: Stretched-exponential exponent *γ* as a function of *ϕ* for all *κ* as in (**A**).

Making the particles more compressible significantly changes this behaviour, and leads to volume adaptation of the particles as the particle packing fraction is increased. Both for intermediate and high compressibilities, *κ* = 500 (Fig. 1B) and *κ* = 50 (Fig. 1C), we observe a strong growth of the polydispersity induced by osmotic deswelling. This is visible in the renderings of the simulation box (Fig. 1B,C) and in the deswelling distributions (Fig. 1E,F,H,I,K,L). Not only does the average particle size shrink as the density is increased, visible as a shift of the mean *a*/*a*_0_ to lower values, we observe a distinct broadening of the distributions. Since the glass offers a heterogeneous micro-environment for each particle, due to the disordered structure, the local pressure each particle experiences is also inhomogeneous such that a broad distribution of deswelling degrees results. This can be most clearly seen in the case of the very compressible particles (*κ* = 50, Fig. 1C). In all cases, we find that the initially bidisperse system remains in a glassy state, with no signs of medium- or long-ranged crystalline order, as evident from a liquid like pair-correlation function *g*(*r*) (Fig. 2A).

These observations of strong local volume regulation in a system of size mismatched compressible particles is in direct agreement with seminal experiments by the groups of Lyon and Fernandez-Nieves; they showed that a single large and soft microgel can exhibit severe deswelling to fit into a crystalline lattice of smaller particles^37,38,48^. In the final section of this manuscript, we will show that our simulation model can in fact reproduce these specific observations.

### Apparent fragility

We proceed to explore the effects of this concentration-dependent osmotic deswelling, regulated by the compressibility energy scale *κ*, in the slowing down of structural relaxations. To do so, we compute the self-intermediate scattering function from our simulation data as ${F}_{s}(q,t)=\langle \exp (i{\bf{q}}\cdot [{\bf{r}}(t)-{\bf{r}}(0)])\rangle$ where we choose $q=2\pi /\bar{a}$ as the scattering vector to probe particle self-diffusion, where $\bar{a}$ is the ensemble-mean particle size for that particular simulation run. The shape of the self-intermediate scattering functions (Fig. 2C) is very similar to those found for two-dimensional colloidal glasses of soft particles, both in experiments^49^ and in simulations^50^, with a characteristic terminal decay that shifts to larger times as the concentration is increased, reflecting the slowing down of structural relaxations. To test the robustness of our simulation with respect to the calculated self-intermediate scattering functions we perform four repeats of three different packing fractions for *κ* = 50000 (Fig. 2B). As we find almost perfect agreement between the replicates — fitted values for the long-time structural relaxation time, *τ*, are within 5% error of each other — we conclude that our simulations are highly robust and repeatable.

While the *F*_*s*_(*q*, *t*) curves do not show a clear separation between a short time and a long time decay, typically interpreted as a fast cage rattling and slow cage breaking motion, we find that they cannot be fitted by a single stretched exponential decay but are described well by a double exponential decay (solid lines in Fig. 2C), indicating that these two modes of relaxation are still present. The smoothing of the two decays into a quasi-continuous and strongly stretched decay may be the result of the long-wavelength fluctuations that exist in two-dimensions. Whether these effects alter the physics of the liquid-solid transition as a function of the dimensionality of the system, is a recent topic of debate^49–52^ and is outside of the scope of this manuscript.

For incompressible particles, *κ* = 50000, the characteristic long-time structural relaxation time *τ*, normalised to that for freely diffusing particles at very low densities *τ*_0_, shows a steep growth at an effective area fraction *ζ* = 0.8 (Fig. 2D, green squares), in accord with experiments^49^. By contrast, lowering the particle compressibility, leads to a much more gradual growth of the relaxation time (Fig. 2D red down-pointing triangles). We observe how values of the effective packing fraction *ζ* well above unity are required to realize a significant slowing down of the dynamics; this is in direct agreement with experimental observations on suspensions of soft and compressible spheres^20^. Whereas in^20^ the authors find a clear dependence of the stretching exponent, *γ*, on the particle softness we find no such dependence; the exponents from the fits for different particle stiffnesses, *κ*, all overlap (Fig. 2F inset).

To define a glass transition point *ζ*_*g*_, we follow the approach employed previously by other authors, as the packing fraction where $\tau /{\tau }_{0}\equiv {10}^{5}$^11,53^. While this definition is somewhat arbitrary by definition, the same approach is used in the study of fragility in molecular glasses, as first proposed by Angell^19^. By direct analogy, we can construct a so-called Angell plot of our simulation data in which we plot the relative growth of *τ* as a function of the scaled packing fraction *ζ*/*ζ*_*g*_.

Indeed we find that this gives rise to apparent changes in the fragility of the colloidal glass transition. Whereas the incompressible disks exhibit a fragile vitrification, the strength of the glass transition increases as *κ* decreases. Interestingly, this result required no a-priori assumption of the shape of the dynamical slowing down nor on the equation-of-state of the suspension, while this was required in our previous analytical theory. This confirms the validty of our theoretical argument explaining how particle compressibility governs the apparent fragility of the colloidal glass transition. This becomes even more clear when we quantify the apparent fragility of the glass transition using the kinetic fragility index, defined for colloidal systems as:

6 $$m={\frac{d\mathrm{log}(\tau /{\tau }_{0})}{d(\zeta /{\zeta }_{g})}|}_{\zeta ={\zeta }_{g}}$$

which has a limiting value for a strong glass $m\equiv 5$, dictated by our definition of the point of vitrification as log(*τ*/*τ*_0_) = 5 and grows as the glass transition becomes increasingly more fragile. In validation of our theoretical result, we find that *m* is directly controlled by the particle compressibility *κ* (Fig. 2E inset). Moreover, the values of the fragility index *m* are in direct agreement with our previous theoretical study.

We have now shown how these coarse-grained simulations capture some of the essential features previously found in experiments^20^ and in analytical theory^21^. However, as speculated in the introduction, the observed fragility changes may only be apparent ones that arise from the use of an extrapolated packing fraction, which is strongly non-linear in the true volume fraction *ϕ*. If this is true, all the curves of *τ*/*τ*_0_ should collapse when plotted against *ϕ*, which accounts for the actual deswelling ratio of the particles and thus is a measure for the real free volume in the suspension. Indeed, we find that all of the simulation data, from very hard and incompressible disks to the most compressible ones explored here, can be collapsed simply by exchange the state variable *ζ* for the true volume fraction *ϕ* (Fig. 2F). This not only confirms that the assumption of universal dynamics with *ϕ* in our previous analytical theory was correct^21^ but that the apparent fragility changes in soft colloidal systems are not associated with a change in microscopic physics.

**Figure 3:**
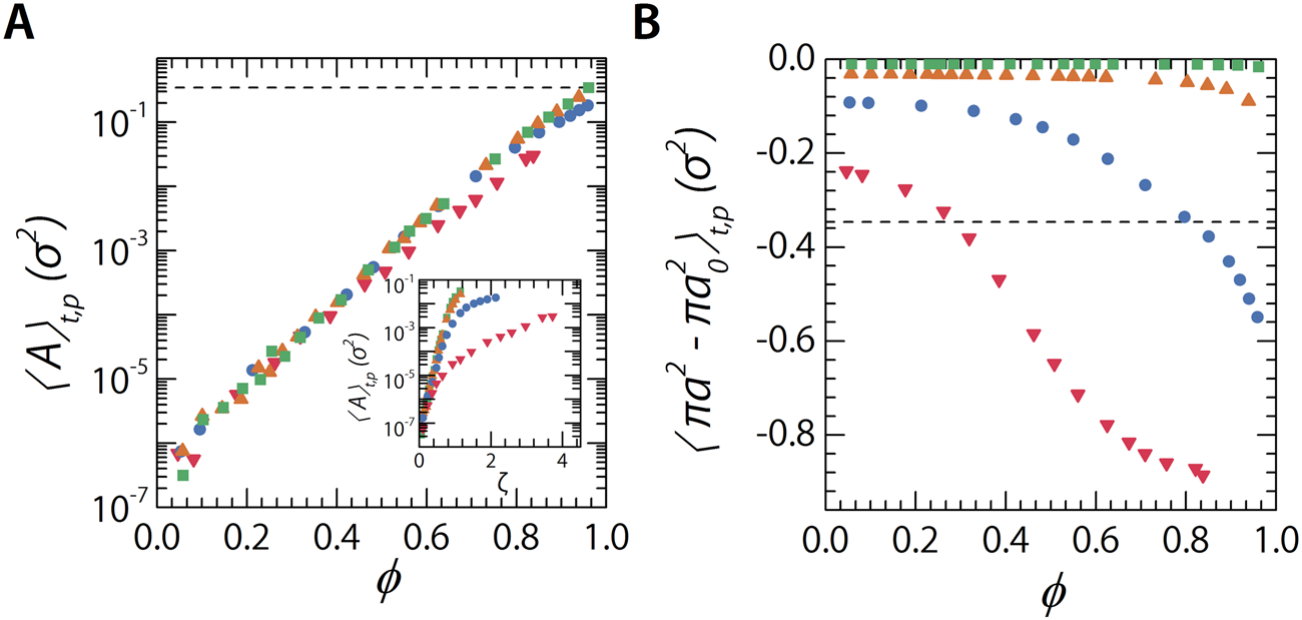
(**A**) Overlap area per particle, averaged over the last 100 simulation snapshots, as a function of *ϕ*. The dashed line indicated the maximum overlap found at 0.34 *σ*^2^. Inset: Same data plotted as a function of *ζ*. (**B**) Average change in area of the particles as a function of *ϕ*. As in panel **A** this is averaged over the last 100 simulation snapshots. Dashed line again indicates a value of 0.34 *σ*^2^.

De-swelling is not the only mode able to change the relation between packing and volume fractions. Experiments show that at very high densities polymeric microgel particles can interpenetrate^54^. The interaction potential used in our studies also allows for some degree of overlap between our particles; to test whether this only occurs at high densities as conform experiments we calculate the overlap area per particle and plot this as a function of *ζ* and *ϕ* (Fig. 3A). We find modest overlaps with a maximum at maximum volume fraction where the overlap area *A* = 0.34. Comparing these findings with the loss of particle area as a result of de-swelling, it is clear that our particles adapt to the increase of concentration predominately via de-swelling with interpenetration only playing a role at very high densities or very high particle stiffnesses (Fig. 3B). This is further corroborated by the observation that the change in particle area is most definitely, for low to moderate *κ*, dependant on particle stiffness. This is expected if it forms the defining adaptation mode for the increase in density.

Previous simulations on systems with different interaction potentials have shown a small dependence of fragility on the polydispersity of the sample under study^55,56^. In our case there definitely is polydispersity, but a major difference should be taken into account: our polydispersity is not constant with *ϕ* and is a fully emergent property of the system in response to an increase in osmotic pressure.

### Softness and inhomogeneous dynamics

So far we have used our minimal simulation model, which accounts at a coarse-grained level for osmotic regulation and particle deswelling, to validate our analytical theory proposed previously and to illustrate the origin of apparent fragility changes in colloidal glasses of soft particles. Recent experiments of Rahmani *et al*.^30^ have hinted that in addition to changing the apparent glass fragility, particle softness also has a marked effect on the emergence of dynamical inhomogeneities. This is an interesting observation since fragility transitions and the propensity of systems to exhibit inhomogeneous local dynamics have also been linked for molecular and atomic glasses^57,58^. We can thus ask whether our simulations give rise to similar observations.

**Figure 4:**
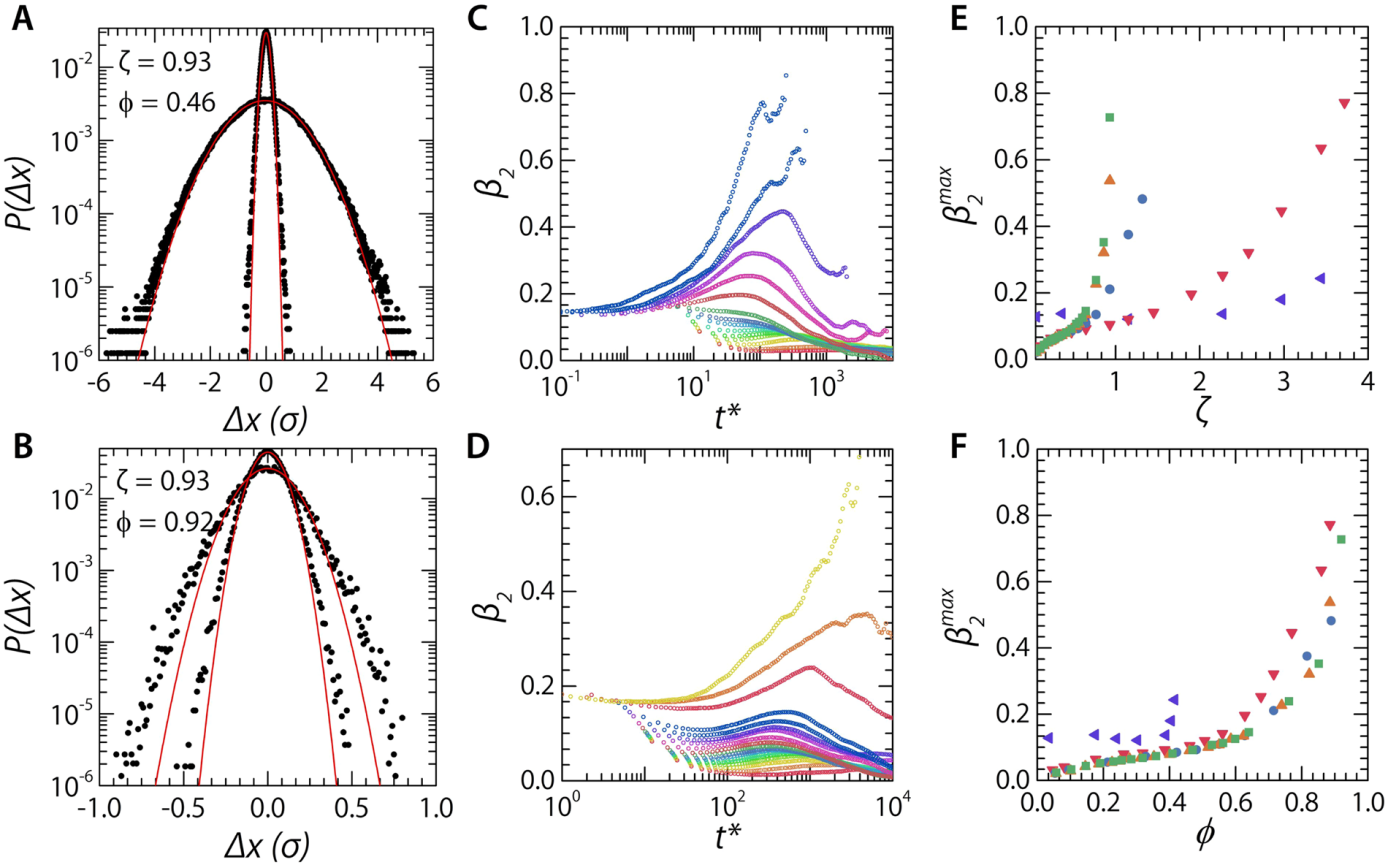
Displacement probability distribution *P*(Δ*x*) from our simulations. For soft particles, *κ* = 50, (**A**) and incompressible particles, *κ* = 50000, (**B**) Both have displacements recorded at dt* ≈ 6 and dt* ≈ 1100. Note the difference in true volume fraction, *ϕ*, between the two samples. (**C**) Non-Gaussian parameter *β*_2_ for soft particles, *κ* = 50 and (**D**) for incompressible particles, *κ* = 50000. As a function of lag time *t* for a wide variety of packing fractions (red = low, blue = high), (**E**) Maximum value of the non-Gaussian parameter ${\beta }_{2}^{max}$ as a function of the extrapolated packing fraction *ζ* and (**F**) as a function of the true volume fraction *ϕ*.

To explore the homogeneity of the dynamics in our simulation model, we use the standard approach often employed for colloidal glasses. First, we compute the displacement probability distribution *P*(Δ*x*(t*)) from our simulations at constant *ζ*. For the soft particles we observe how the step size distribution is relatively Gaussian at small value of t*, and stays this way for larger values of t*. For the hard particles we see how longer t* gives rise to a distribution with exponential tails at large displacements (Fig. 4A,B). The difference between the two can be found in the fact that even though the samples are at equal *ζ* their effective volume fractions are widely different, *ϕ* = 0.46 versus *ϕ* = 0.92. The exponential tails are the signature of a wide distribution of local mobilities, a characteristic fingerprint of the glassy state, and have been observed also in experiments on colloidal glasses^4,59^.

To further elaborate on the inhomogeneous dynamics of the system, we can compute the non-Gaussianity of the displacement distributions as the ratio of the second and fourth moment of the distribution:

7 $${\beta }_{2}(t)=\frac{\langle {\rm{\Delta }}{\tilde{x}}^{4}\rangle }{3{\langle {\rm{\Delta }}{\tilde{x}}^{2}\rangle }^{2}}-1$$

which for a purely Gaussian distribution *β*_2_ = 0.

When we plot *β*_2_ as a function of the correlation time *t* (Fig. 4C,D) we find curves that exhibit a very similar trend as observed previously in experiments, both for two- and three-dimensional colloidal glasses. We note, that at short times *β*_2_ takes a finite non-zero value, while it should tend to zero for a monodisperse system. Here, the non-zero value of the non-Gaussianity originates from the strong polydispersity in our system, leading to a short-time displacement distribution which is a convolution of many Gaussian functions for each particle size population. At larger times, the step size distribution is governed by the particle-particle interactions, and the polydispersity is less relevant, thus probing the inhomogeneous dynamics due to local variations in the micro-environment.

At low volume fractions, the dynamics are very homogeneous and no significant heterogeneity is observed. As the volume fraction is increased and the glass transition point is approached, dynamical heterogeneity emerges as a distinct peak in *β*_2_(*t*). The peak height ${\beta }_{2}^{max}$ can be taken as a proxy for the extent to which the dynamics are inhomogeneous. Plotting these data against the extrapolated packing fraction again appears to indicate distinct differences between hard and soft particles, which much lower values of ${\beta }_{2}^{max}$ for a given value of the packing fraction *ζ* (Fig. 4E). However, also here we can ask if these differences are artificial due to the fact that the system dynamics are governed by the true volume fraction. Replotting ${\beta }_{2}^{max}$ as a function of *ϕ* reduces the differences between the different softnesses to a large extent (Fig. 4F). This suggests that, in analogy to the fragility, also changes in the amplitude of dynamical heterogeneities vanish when the appropriate state variable *ϕ* is used. We note that in this case plotting the data as a function of *ϕ* reduces the difference between the curves to a large extent but does not collapse the data sets perfectly. In particular for the softest particles, we find stronger non-Gaussian displacements at lower volume fractions as compared to their incompressible counterparts. This may be due to the fact that the softest particles show the largest extent of osmotic deswelling, which leads to a strong increase in the polydispersity of the suspension as shown in Fig. 1. If the particle size fluctuates significantly under conditions of strong osmotic regulation, i.e. small values of *κ* and high volume fractions *ϕ*, the time-averaged value of the particle size *a*_*i*_ that we use to account for polydispersity in computing $P({\rm{\Delta }}\tilde{x})$ may not be able to fully correct for particle size disparity.

**Figure 5:**
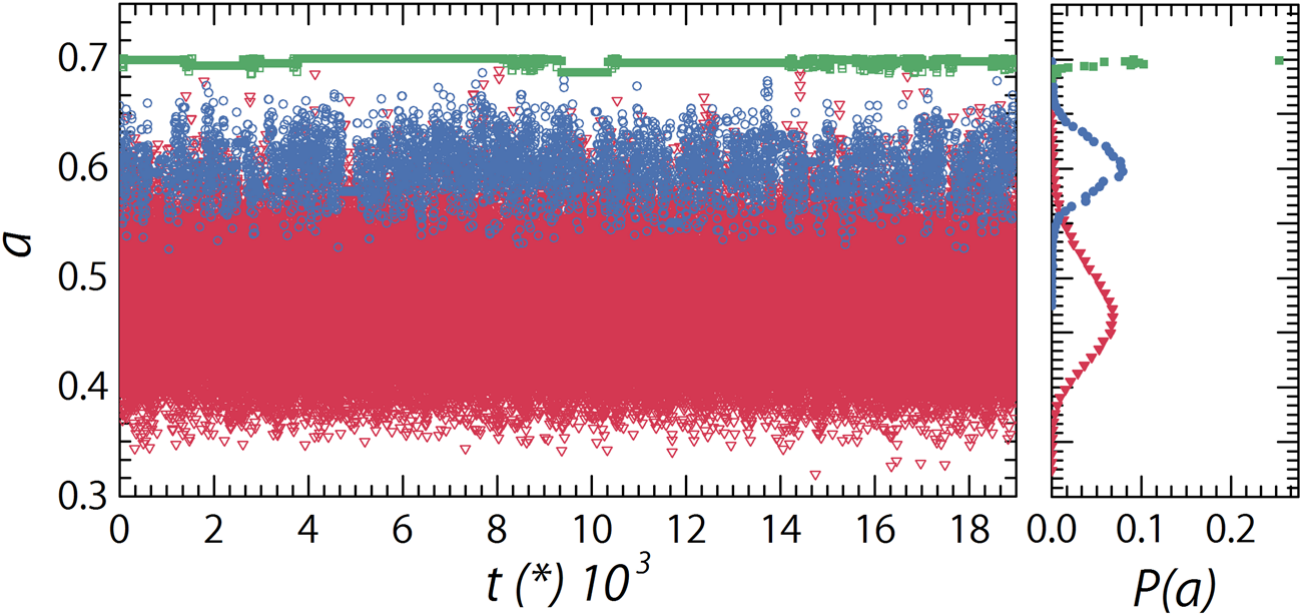
Left: Osmotically-induced particle size fluctuations as a function of time, for *κ* = 50 (red triangles), *κ* = 500 (blue circles), and *κ* = 50000 (green squares). Right: corresponding probability distributions *P*(*a*) of the size of a single particle over time, which reveal a Gaussian shape. All samples are at *ζ* = 0.93.

To highlight this effect, we plot the temporal fluctuations in the size of a single particle *a*(*t*). As the glass is dynamic, the local microenvironment a particle experiences fluctuates in time, leading to changes in the local osmotic pressure. For hard particles, whose size is relatively independent of the local pressure, the size is thus also constant in time with only minor fluctuations (green square symbols in Fig. 5). By contrast, for softer particles, the fluctuations in the microenvironment lead to strong size fluctuations (blue circle and red triangle symbols in Fig. 5). We also note that the distribution *P*(*a*) of temporal size fluctuations is Gaussian in all cases (right panel Fig. 5). Due to the large fluctuations for the softest particles, correcting for these by a time-average value of 〈*α*_*i*_〉 to obtain the size-corrected displacements ${\rm{\Delta }}{\tilde{x}}_{i}$ is thus only a first order correction and presumably leads to the small deviations in the collapse of the curves of the dynamical heterogeneity versus true volume fraction *ϕ* (Fig. 4F). On the basis of these data we thus conclude that the imperfect collapse of the data in Fig. 4F is not due to real changes in the nature of dynamical inhomogeneities.

**Figure 6:**
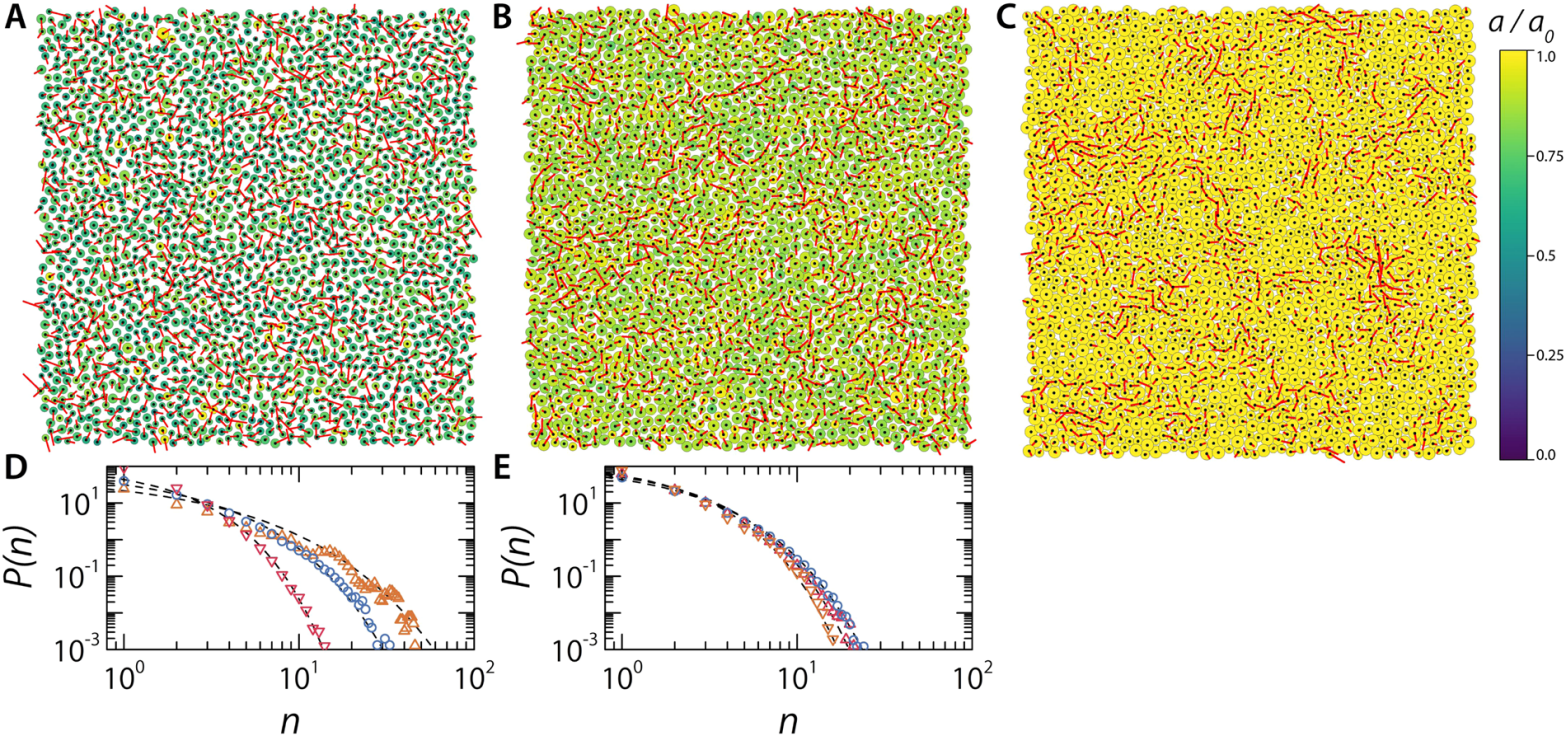
Snapshot from the simulations with the particles color coded according to their *a*/*a*_0_, with their displacements $\delta \overrightarrow{r}$ over a time interval equal to the lag-time of ${\beta }_{2}^{max}$ projected as a scaled red line, for (**A**) *κ* = 50, *ζ* = 0.93 (*ϕ* = 0.46), and dt* = 71 at 1.5x original displacements (**B**) *κ* = 500, *ζ* = 0.93 (*ϕ* = 0.71), and dt* = 529 at 1.5x original displacements (**C**) *κ* = 50000, *ζ* = 0.93 (*ϕ* = 0.92), and dt* = 4180 at 2.5x original displacements. (**D**) Probability distribution of cluster size for cluster made up of the 10% most mobile particles. Calculated for three samples at the same *ζ* and *κ* = 50, 500 & 50000 corresponding to panels (A–C). Dashed lines are guides to the eye (**E**) Probability distribution of cluster size for cluster made up of the 10% most mobile particles. Calculated for three samples at similar volume fractions, *ϕ* = 0.63, 0.63 & 0.64, and *κ* = 50, 500 & 50000. Dashed lines are guides to the eye.

Finally, we confirm this by inspecting the spatial configuration of particle displacements under conditions where dynamical inhomogeneities are significant. We reconstruct our simulation box at a given point in time by colour-coding the particles according to their relative size, *a*/*a*_0_, and superimposing their instantaneous displacement vector *δ*$\overrightarrow{r}$ over a time interval dt* equal to the time at which we find the maximum in *β*_2_ (Fig. 6); we observe that in the case of identical *ζ*, the softest particles do not show dynamical heterogeneity, while pockets of correlated particle motion emerge for stiffer particles in a background of largely immobile colloidal glass. To further quantify these findings we perform a cluster analysis: calculating the cluster size probability distribution on the 10% most mobile particles in each sample, based on a distance cut-off equal to the first minimum of the radial distribution function for that specific sample. As the particle stiffness increases we observe the appearance of clusters of highly mobile particles, a hallmark feature of dynamical heterogeneity (Fig. 6D). We assume that dynamical heterogeneity also collapses as a function of *ϕ* and the analysis of these clusters does appear to support this. If we analyse samples at highly similar volume fraction, *ϕ* = 0.62–0.63, instead of similar packing fractions *ζ,* we observe a full collapse of the cluster size probability distribution (Fig. 6E).

These data thus imply that both the changes in fragility and the changes in dynamical inhomogeneity as a function of softness that have been observed in experiments^20,30^ are only apparent ones that result from the overestimation of the packing fraction when using *ζ* rather than *ϕ* as the experimental control parameter.

### Crystals and selective deswelling

Above we have shown how our minimal simulation model can account for the softness-dependent osmotic deswelling of compressible colloids in dense suspensions and how this may lead to apparent changes in the microscopic properties of colloidal glasses made from these particles. The effect of softness in colloidal suspensions extends far beyond the realm of disordered glasses and has a pronounced effect on a wide variety of phenomena, including crystallisation and supramolecular self-assembly. Our simulation model can be readily extended to explore how deswelling influences these scenarios as well.

As a proof-of-principle we consider the crystallisation of monodisperse microgel particles in which a small fraction of larger impurities is introduced. This scenario was explored both when the particle species are of equal stiffness^60^ and in cases where the larger impurities were effectively softer, e.g. due a crosslinking gradient^37,38,48^. In both cases, the size ratio of small to large particles in their swollen state was ~0.6–0.7. In the first case, it was found that a small amount of impurities can be accommodated by the crystal lattice of the smaller particles by creating a substitutional vacancy defect, whereas impurities above a certain threshold concentration created a bidisperse glass^60^. In the second case, a selective deswelling of the softer large particles was observed. In this scenario, the osmotic energy penalty for selectively deswelling the large particle is apparently balanced by the energy gain through reduction of lattice strains in the crystal. It was recently shown that the selective deswelling of larger particles in a sea of smaller ones can be explained on the basis of the overlap of the diffuse cloud of counterions at high enough packing densities in charged suspensions^37,48^. By adapting our approach to the case of a large impurity in a crystal of smaller ones, we will show here that a softness mismatch of sufficient strength, between impurity and matrix particles, is sufficient to achieve selective deswelling and obtain a defect-free crystal despite the size disparity.

**Figure 7:**
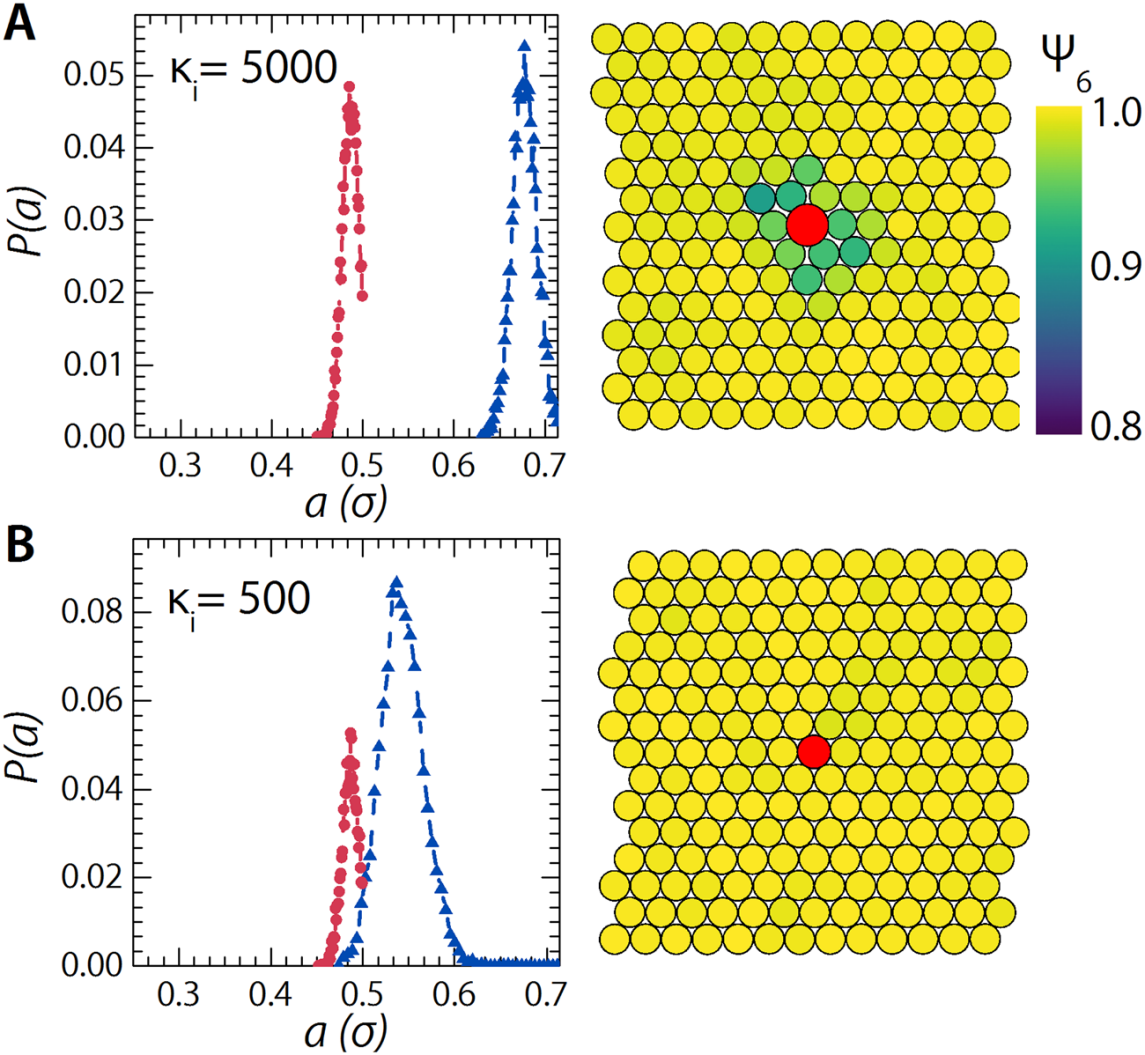
Left: Probability distributions *P*(*a*) of the size of a single particle over time for a particle in the crystalline matrix far away from the intruder (red circles) and for the intruder (blue triangles). Calculated from simulations with *κ*_*i*_ = 5000 ((**A**) equal to the matrix) and *κ*_*i*_ = 500 ((**B**) weaker than the matrix). Right: corresponding *ψ*_6_ maps, calculated over the average of 1000 consecutive simulation snapshots. The maps show a small fraction of the simulation box (*N* = 2025) centred around the intruder particle depicted in red. All samples are hexagonal close-packed at initiation of the simulation.

We initialize our simulation model by creating a two-dimensional crystalline initial configuration of monodisperse particles of *a*_*S*,0_ = 0.5*σ*. We introduce a single substitutional impurity by replacing one particle with a large impurity with *a*_*L*,0_ = 0.71*σ*, which gives a size ratio of 0.7. The small matrix particle have a fixed compressibility energy of *κ* = 5000. If we introduce a substitutional impurity with the same compressibility as the matrix, *κ*_*i*_ = 5000, we find that the impurity distorts the surrounding crystal lattice and causes the proliferation of defects (Fig. 7A). We visualise this effect via a time-averaged positional map, colour coded according to the two-dimensional order parameter, ${\psi }_{6,i}={n}_{c}^{-1}\,{\sum }_{j=1}^{{n}_{c}}\,\exp \,[i6\theta ({r}_{ij})]$, where *n*_*c*_ is the number of neighbours of particle *i* over which the sum runs.

Clearly, introducing multiple of such impurities would result in a loss of crystal symmetry and result in a bidisperse glass at sufficient impurity levels. This is in accord with experimental findings^60^. However, if we make the impurity significantly softer than the surrounding matrix, *κ*_*i*_ = 500, we observe that it indeed deswells selectively, reducing the amount of defects and the spatial extent of the ‘damage’ zone, until for the softest impurity it accomodates fully to the lattice resulting in a defect free crystal (Fig. 7B). These results suggest that impurities of identical compressibility to the matrix will form glasses at sufficient packing density^60^, while a compressibility mismatch can result in selective deswelling with the crystalline order retained despite the difference in the fully swollen size^37,48^.

Due to the method by which microgel colloids are typically prepared, resulting in a radial gradient in crosslinking density from the particle core to its periphery^61^, larger particles are somewhat softer than smaller ones when prepared with identical initial crosslinker to monomer ratio. As a result, even in polydisperse microgel suspensions prepared in the same pot, this selective deswelling may reduce the particle size dispersity at high packing fractions, thus allowing such compressible particles to crystallize at higher initial polydispersity as compared to hard and incompressible spheres, where high monodispersity is required to form defect free crystalline structures^62^.

## Conclusion

We have shown how particle size regulation can be taken into account in a dynamical Monte Carlo model for soft and compressible colloids. This enables the study of osmotic deswelling in dense suspensions. We find that for colloidal glasses of compressible colloids apparent changes in the liquid fragility and dynamical heterogeneity emerge due to the use of an extrapolated packing fraction as the control parameter, while the dynamics are universal when plotted as a function of the true volume fraction, which takes the deswelling into account. This suggests that colloidal glasses of soft particles are always fragile when the appropriate state variable is used. Moreover, we show how the same method can be used to explain the selective deswelling of a substitutional impurity in a colloidal crystal of microgel particles. While coarse-grained in nature our method enables the efficient simulation of the effects of osmotic deswelling in dense systems of soft and solvent-swollen colloids. In this manuscript we have explored the effect this has on phase transitions in purely repulsive systems, but in principle the method can be readily adapted to additionally explore the effects of volume regulation on the self-assembly of soft colloids either driven by external fields^63,64^ or by means of supramolecular interactions^65–67^.
